# A Novel Prognostic Model Using Chaotic CNN with Hybridized Spoofing for Enhancing Diagnostic Accuracy in Epileptic Seizure Prediction

**DOI:** 10.3390/diagnostics13213382

**Published:** 2023-11-03

**Authors:** Preethi Palanisamy, Shabana Urooj, Rajesh Arunachalam, Aime Lay-Ekuakille

**Affiliations:** 1 Department of Computer Science and Engineering, Kongunadu College of Engineering and Technology, Trichy 621215, India; 2 Department of Electrical Engineering, College of Engineering, Princess Nourah bint Abdulrahman University, P.O. Box 84428, Riyadh 11671, Saudi Arabia; 3 Department of Electronics and Communication Engineering, Saveetha School of Engineering, SIMATS, Chennai 602105, India; drrajesha86@gmail.com; 4 Dipartimento d’Ingegneria dell’Innovazione (DII) (Department of Innovation Engineering), Universita del Salento (University of Salento), Via Monteroni, Ed. “Corpo O”, 73100 Leece, Italy

**Keywords:** seizure, EEG, neurodegenerative, transfer learning, fatal risk, optimization and chaotic CNN

## Abstract

Epileptic seizure detection has undergone progressive advancements since its conception in the 1970s. From proof-of-concept experiments in the latter part of that decade, it has now become a vibrant area of clinical and laboratory research. In an effort to bring this technology closer to practical application in human patients, this study introduces a customized approach to selecting electroencephalogram (EEG) features and electrode positions for seizure prediction. The focus is on identifying precursors that occur within 10 min of the onset of abnormal electrical activity during a seizure. However, there are security concerns related to safeguarding patient EEG recordings against unauthorized access and network-based attacks. Therefore, there is an urgent need for an efficient prediction and classification method for encrypted EEG data. This paper presents an effective system for analyzing and recognizing encrypted EEG information using Arnold transform algorithms, chaotic mapping, and convolutional neural networks (CNNs). In this system, the EEG time series from each channel is converted into a 2D spectrogram image, which is then encrypted using chaotic algorithms. The encrypted data is subsequently processed by CNNs coupled with transfer learning (TL) frameworks. To optimize the fusion parameters of the ensemble learning classifiers, a hybridized spoofing optimization method is developed by combining the characteristics of corvid and gregarious-seeking agents. The evaluation of the model’s effectiveness yielded the following results: 98.9 ± 0.3% accuracy, 98.2 ± 0.7% sensitivity, 98.6 ± 0.6% specificity, 98.6 ± 0.6% precision, and an F1 measure of 98.9 ± 0.6%. When compared with other state-of-the-art techniques applied to the same dataset, this novel strategy demonstrated one of the most effective seizure detection systems, as evidenced by these results.

## 1. Introduction

Common neurodegenerative ailment seizures, defined as constantly reoccurring, cause human brains to behave quickly and improperly. This illness is brought upon by aberrant neuronal brains, according to one source [1]. Seizure production involves an abnormal surge of electrical activity in the brain due to increased neuronal excitability and an imbalance between excitatory and inhibitory signals. This imbalance leads to synchronized and excessive firing of neurons, resulting in the characteristic symptoms of a seizure. Various factors, including genetics, brain injuries, and chemical imbalances, contribute to the disruption of normal brain activity, triggering seizures. Interruptions of physical, sensorial, behavioral, and even the importance of creativity frequently accompany epilepsy brought on by this uncontrolled neural burst [2]. Every victim experiences aberrant responses after acute neuronal condition arises, including dizziness, bodily imbalance, seizures, involuntary spasms, unconsciousness, and even insight [3]. Symptoms may have a significant negative effect on each part of the victim’s body and their family’s safety, as well as being a potential risk for anyone who has a fatal risk. Seizure illnesses have long been shrouded with stigmas, misinformation, prejudice, and even panic. The stigma that surrounds the illness still exists in several nations and affects the standard of living for those with this condition, not to mention their families, too [4]. Seizures are among the more prevalent brain disorders globally, affecting over half a billion patients [5]. Approximately 81 percent of overall survey seizures affected patients, and many people from poor as well as moderate-income nations do not receive the necessary care [4]. Epilepsy is manageable. Generally, pro medications could today centralized area epileptic that represents gift again such majority of individuals who suffer from the disorder. It is currently possible to cure neurological affection, and it significantly affects sufferers’ lives [6]. In this outcome, timely epileptic diagnosis becomes necessary for overall seizure treatment. Accurate diagnosis for epileptics is important because it gives the patient time to seek routine care to help manage their hallucinations plus avoid locations that might heighten their vulnerability while they are occurring [7].

This ability to anticipate seizures earlier is crucial for sufferers, their relatives, and medical professionals. (Electroencephalography) EEG and electron microscopy, with neuroimaging, are just two of the diagnostic methodologies that detect epileptics [8]. Compared to the other techniques, electroencephalography is regarded as the most viable way of constantly recording excitability and subtle signs of brain diseases. Dual parts of EEG are the scalp (EEG) as well as intracranial (iEEG) [9]. EEG records non-invasive developments compared to costly iEEG, which offers numerous benefits while being better accepted. Only as a result, hairline currently, EEG is the most robust approach to identifying and forecasting epilepsy [10]. 

**Figure 1:**
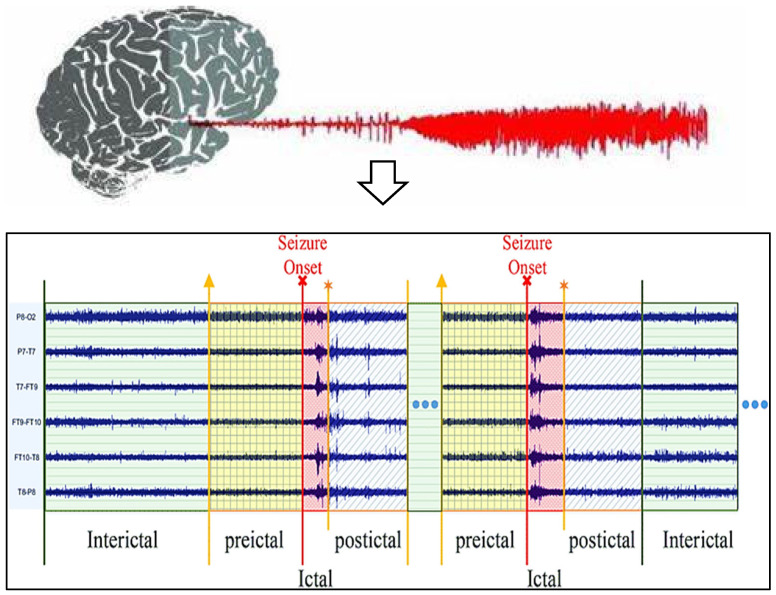
EEG activation levels of patients with epilepsy (Yellow arrow: Starting state of preictal, Red arrow: Starting state of ictal, Orange arrow: Starting state of postictal).

Data analysis technologies have become increasingly popular, and many have been drawn toward utilizing them. Machine learning and deep learning are used in information retrieval information theory in various disciplines [11] due to the rapid expansion of digital technologies, and these improvements are microbially cognitive. Several earlier investigations indicate it may be feasible to forecast spontaneous epilepsy despite the absence of a complete theory as well as data on the cause of epileptic fits [12]. The electroencephalography epilepsy estimation technique is also becoming more accessible while EEG records are getting better. Despite the considerable improvements noted in this paper, there are certain difficulties with EEG-based seizure detection. This use of predictive change starts initially. Seizure predictions are primarily used to treat symptoms rather than avert infections, which requires giving that sufferer enough time to get ready for impending epileptic fits. This recent research has concentrated on pre-ictal, preictal, and interictal range predictions through the real picture of seizure frame level, demonstrated within the EEG activities phases for seizure, as shown in Figure 1. This requirement for an efficient seizure diagnosis system to adhere to basic parameters represents a main trial. Epileptic prognosis is currently in this phase of conceptual discovery despite its demand for medical trials. Because of this significant amount of complexity in EEG data, this evaluation of seizure phenomena presents this final difficulty [13]. Its exploration of both seizures and preictal impulses is mainly concentrated in the current studies about subjects; therefore, choosing suitable techniques for its analyses has currently significant scientific work [14].

Seizures are abrupt and temporary disruptions in the brain’s electrical activity, causing diverse physical and mental symptoms. Epilepsy, on the other hand, is a neurological disorder marked by recurrent, unprovoked seizures resulting from factors such as brain injury, genetics, or unknown causes. While seizures are the characteristic events of epilepsy, the latter term encompasses the broader chronic condition characterized by the tendency to experience such seizures. Hence, in this research, a semi-head electroencephalography approach for seizure as well as epilepsy detection is proposed. Epilepsy predictions were accomplished using deep learning techniques for categorizing the many time frames that make up or before the interval. To represent the data embedded in the EEG recordings, a seizure prediction system using gated recurrent units (GRU) and deep residual shrinkage networks (DRSN) is created. This system includes smooth edge detection signals denoising but also attentive mechanisms. 

The article is structured as follows: the second chapter covers an earlier study on epileptic detection, the third chapter details suggested techniques, the fourth chapter presents research observations with analysis, and the fifth chapter provides a working summary with a forecast.

## 2. Related Work

There are three major epileptic stages, as per Figure 1. The epileptogenic plus period-ended stages of the EEG can be particularly helpful with predicting epilepsy episodes. Samples for seizures and non-seizures are distinguished using the onset stage. A further useful stage in the identification of epilepsy is the transition between the two stages. This starts a very few seconds before a seizure starts but stops when the epileptic stage starts. Various specialists [15,16,17,18,19] have also attempted to use brain activity to identify the onset of both the prodromal states. Some have successfully identified this prodromal condition in epileptics. Accurate involuntary spasm detection depends on image retrieval with pretreatment of EEG recordings to improve transmitter levels. 

That interictal condition underlying epilepsy is predicted more by integration among several distinctive markers towards the classification model. Using multivariate regression with limited flexibility, the devised method was used for detecting seizures. The same researchers retrieved twenty-two univariate regression features from our conceptual scheme using just six electroencephalographic signals. Therefore, a subspace with 132 dimensions is produced. These were considered than interictal treatment ten-forty min’s variation between ten min-before an epileptic stage. With divided testing, this dataset has two categories: the preictal phase and the epileptogenic stage. A Boolean classification that categorizes the training dataset can be utilized for detecting psychotic episodes. Following those techniques, 74 percent accuracy was predicted. 

Researchers have employed SVM for classifying EEG channels between the preictal and ictal stages. During this approach, the researchers utilized obtained multivariate regression characteristics with one predefined threshold of 10 ms. After some normalization plus pretreatment inside Stage 2, a design for that electroencephalogram was chosen. In addition to applying devices here to suffer a person’s head and focus upon status epilepticus, three electroencephalographic signals were retrieved. All three devices were situated beyond a seizure region. Information from recorded electroencephalogram recordings is divided into parts using five five-second uncorrelated frames. Its sound influence was minimized by applying the modulation scheme [20] following the translation of these input five-second segments. 

The first four statistical instances that were retrieved are traits detected by the researcher. These qualities not only simultaneously assess overall parallelism and variation but also uniformity for successive electroencephalogram channels. These writers standardized characteristics that prevent exceptions in an attempt to deal with and manage them. Even brains seem to have a stochastic medium of receiving baseline electrical activity. There is a little distortion inside these readings despite efforts to decrease it. These samples are smoothed throughout this goal of removing distortion. 

Predictor variables regression elements were shown to be more effective during the research when used to analyze epileptic symptoms from channels of EEG. While selecting six electroencephalogram signals and extracting twenty-two linear unitary variables to every signal, In suggested a system to detect epileptic convulsions. This featured an area’s entire specifications increase to one thirty-two. Also, six sensors were employed by researchers to collect extracted features. Since patients find it uncomfortable having several monitors over their skull, the primary goal of such minimal sensor selections could help improve patient comfort without the need for such a high assortment of capacitors. Consequently, only six networks were purchased as well as utilized for detection to ease the patient’s comfort. These devices were chosen by researchers utilizing three varieties of techniques. 

This first strategy includes choosing six electroencephalography sensors arbitrarily. In contrast, this step involves choosing information propagation with the asset for generating upon that part of that same skull wherever epilepsy begins. This function generator [21] was employed more by researchers as a segmentation phase for smoothness before denoising. Researchers also tried out various transfers plus four main prodromal stage intervals to evaluate the detection performance in its algorithm. Researchers had detected each seizure approximately for the purpose of categorization. Following the selection of appropriate characteristics, trained elements are again put through SVMs to create an organization, whereas performance datasets are instead passed and assess categorization precision as well as reliability. Outside of 88 epileptic fits, the research proposed framework recognized an accuracy of 75.8 percentage points for prediction. These researchers have stated that additional results will enhance efficiency.

Any algorithms for anticipating epileptic episodes could help people with this condition live longer. Researchers retrieved signal strength characteristics, which are then fed through SVMs for categorization since being appropriately chosen. The authors found effective adaptability was 75.8 percent, which implies that even 68 of 88 convulsions were detected by using this classification system. Researchers concluded that by employing such techniques, reduced key frame selection could fare better on feel-like having. These researchers employed the Fourier technique to detect seizures. Curvelet instability as momentum includes the variables we had identified. During evaluation, two or three signals are chosen to diagnose suffering individuals. According to reports, responsiveness is 88 percent, and the mean expectation length is 23 s. This prediction method for convulsions utilizing forehead electrical activity of the brain based upon passband has been put out by. The researchers calculated the distribution of every period inside partial autocorrelation windows but chose averages of occurrences within specific buckets. Following overall completion, the most recent five minutes of measurements were matched to several standard datasets that include prodromal through preictal stages. Researchers established the relevance score of discretized probabilistic random mixture models on ECG signals [22]. Following the calculation of this information utilizing a specific limit, the composite resemblance score was measured. Following the detection of the beginning of said interictal stage, which predicts eventual seizures, another emergency is produced. These scientists used a database with 100 cases and 96 convulsions to test this algorithm in electroencephalogram detection. Researchers have found that now, this system’s sensitivity is 88.3 percent. In this case, the median time it took for anticipating a prodromal condition after seizures began was only 22.6 min. Table 1 compares various deep learning approaches for the number of individuals, epileptic events, EEG recordings, susceptibility, and negative rates.

Researchers have not yet developed a reliable method for transmitting data utilizing brain activity. They present an efficient machine learning strategy for predicting seizures, taking into account that pretreatment of brain activity may enhance detection sensitivity and anticipated period.

## 3. Materials and Methods

To summarize the preliminary results of the study work, this section presents the techniques for the chaotic baker mapping, Arnold transformation, and Teager–Kaiser energy operators.

### 3.1. Method for Signal Analysis

Tegaer–Kaiser energy operator (TKEO) is a nonlinear force monitor operation in the non-stationary signaling process. It can validate the immediate energy of a non-stationary transmission. The frequency and immediate intensity of a message may be monitored using TKEO. HTs separate phases and amplitudes over just a wide range; however, TKEO can identify limited frequency fluctuations. For a continuous signal *g*(*t*), the TKEO is [23].

(1) $$\Psi (g(t))=\dot{g}(t)-g(t)\vec{g}(t)$$

where, *g˙*(*t*) *g¨*(*t*) are the derivative.

Modifying components with time differences yields TKEO for a discontinuous series *g*[*n*] [24].

(2) $$\Psi (g[n])=g^{2}[n]-g[n-1]g[n+1]$$

Exactly three samples are required by Equation (2) to calculate the energy of a message at any given time. As a result, the energy operators have a few characteristics that make them effective in pattern recognition applications, especially voice processing. These characteristics include a brief period, ease of use, and accessibility. In this study, TKEO is used to differentiate between seizures and regular EEG patterns [25].

### 3.2. Arnold and Chaotic Baker Map

The Arnold technique reduces storage and transfer by encrypting the procedure’s maximum number of iterations and altering the pixel’s location. The chaotic approach, which is far more effective, uses permutation-based encrypting to withstand circuit imperfections superior to permeability ones. Throughout this research, the dataset is sent into a reference signal, and the resulting images are encoded to use a stochastic approach. The permutations technique was chosen because it works well in a loud background and is less vulnerable to assaults [26,27].

(3) B(x,y)=(2x,y/2) when 0⩽x<1/2

(4) B(x,y)=(2x−1,y/2+1/2) when 1/2≤x≤1

**Figure 2:**
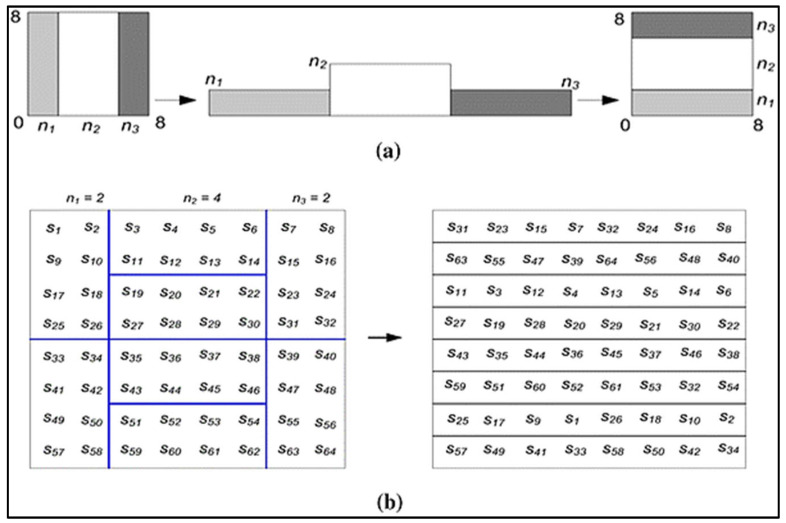
The use of 8 × 8 matrices for Baker mapping. (**a**) Generalized maker (**b**) Distributed Baker.

The following are possible examples of the generalized and discretized baker maps shown in Figure 2. The following generalization applies to the baker map: An *N* × *N* square framework has (M) vertical geometric shapes of *N* being the height and *ni*, being the width wherein *N* = *n*_1 + *n*_2 + … *n*_k should be those vertical rectangles. 

Baker map: Organized into (M) vertical rectangles with height *N* and width *ni*, and *N* × *N* squared matrices. After that, each vertically rectangular with size *N* × *ni* is separated into *n_i_* containers, that each has *N* nodes. Every box is transferred column to a row of images, with both the left one situated at the bottom as well as the right one located at the high. The fractional order mapping formulae are:

(5) $$B_{\left(n_{1},\cdots ,n_{k]}\right.}(r,s)=\left[\frac{N}{n_{i}}\left(r-N_{i}\right)+smod\left(\frac{N}{n_{i}}\right),\frac{n_{i}}{N}\left(s-smod\left(\frac{N}{n_{i}}\right)\right)+N_{i}\right]$$

### 3.3. Convolutional Neural Networks (CNN) Model

**Figure 3:**
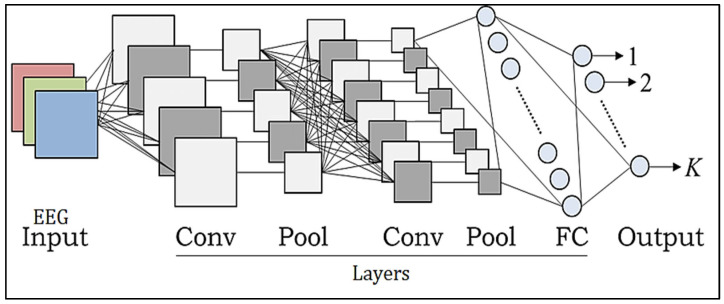
Chaotic CNN core structure.

CNN models increasingly use deep learning algorithms because of their improved performance in object recognition, image analysis, separation, recognition, and NL recognition. CNNs have made significant contributions to the healthcare industry. Classification methods may be used as an efficient automated tool to assist medical professionals in the diagnosis of several disorders. Due to the several feature extraction phases used to successfully achieve the connectivity and localization of the input information, CNN has a strong learning capacity. The CNN design includes replacement convolution and pooling stages, as well as at least one fully linked tier at the conclusion. The combination of CNN components plays a crucial role in creating novel design and construction and, as a result, achieving more desired overall results. This short article briefly describes how these elements fit into the CNNs [28,29]. Prior to CNN, the most often used architectures were AlexNet, Darknet-19, GoogLeNet, ResNet-50, and SqueezeNet [30]. Figure 3 illustrates the architecture.

New indexes of the information sequence at $B_{\left(n1,n2\ldots \ldots ,nk\right)},(r,s)$ are in this example $\left(r,s\right),N_{i}⩽r<N_{i}+n_{i},0⩽s<N$ and $N_{i}=n_{1}+n_{2}+$ $\ldots +n_{i}$.

- Convolution layer:

The convolution layer is made up of the kernel, which is symmetrical. The multiplication is performed as a correlated procedure. The main image was separated into small portions by the convolutional kernels (referred to as regions of interest). The feature extraction process is aided by this division procedure. The kernel processing element containing images employs a particular weighting scheme by multiplying the relevant elements with the responsive field’s multiplication elements. The following equation may be used to estimate convolutional.

(6) $$f_{l}^{k}(p,q)=\sum_{c}\;\sum_{x,y}\;i_{c}(x,y)\cdot e_{l}^{k}(u,v)$$

(7) $$f_{l}^{k}(p,q)=\sum_{c}\;\sum_{x,y}\;i_{c}(x,y)\cdot e_{l}^{k}(u,v)$$

Sliding kernels with the same weighting on images extract distinct sets of attributes making CNN parameters more effective than fully linked networks. The kind and size of filtering, the type of buffering, and the directions of multiplication may all be used to further categorize convolutions.

b. Pooling layer:

An element of CNN is max pooling. Feature motifs, which might occur in multiple places across the picture, are the result of the processing stage. The main purpose is to progressively reduce the number of calculations and factors. As a result, it is also known as downsampling.

(8) $$Z_{l}^{k}=g_{p}\left(F_{l}^{k}\right)$$

$Z_{l}^{k}$ is the max pooling of $I^{h}$ levels for *k*th input data $F_{l}^{k}$, wherein $g_{p}0$. specifies the pool’s procedure.

c. Activation function:

This node ends or connects the NNs classifier. The selection of an appropriate perceptron helps hasten the acquisition. Rectified logistic activation (ReLU), logistic (Sigmoid), and hyperbolic tangent are a few examples of training algorithms. Since it is simple to use and effective at outweighing the limitations of other current training algorithms, such as sigmoid and tanh, it is less vulnerable to a disappearing gradient, which prevents algorithms from being educated. Equations (below) specify the kernel function for a convolution layer:

(9) $$T_{l}^{k}=g_{a}\left(F_{l}^{k}\right)$$

$P_{l}^{k}$ is the convolution outputs that are associated with the kernel function $g_{a}()$. in that case ().

d. Fully connected structure:

This component makes up the network’s last few levels utilized for information categorization. After being flat, the result of the sharing or decoding level is delivered into the connected layer.

### 3.4. Encrypting EEG-Based Chaotic Map

**Figure 4:**
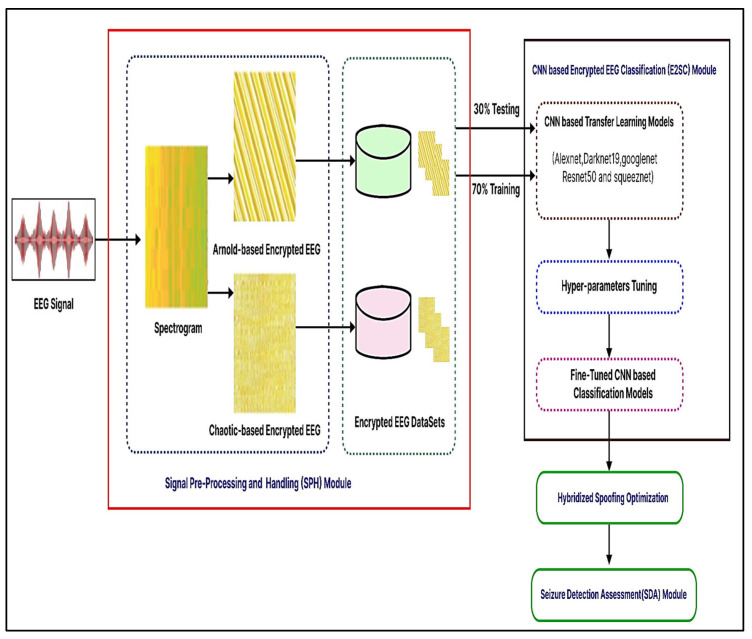
Suggested encryption EEG categorization for epileptic seizures that is CNN-based.

The suggested system is described in this section. The recommended automated encryption EEG spectrogram model’s first steps are shown in Figure 4. There are three different main parts to it, consisting of three modules: (1) Signal Pre-Processing and Handle, (2) Encryption EEG Spectrogram Classifier, and (3) Seizure Detector Analysis. These units are described in the following ways.

#### 3.4.1. Module 1: Pre-Processing Signals

First, a starting point is created in the suggested process to minimize dimensionality by selecting the best factors identified on variable characteristics. The EEG data is then transformed into a 2D spectrum analyzer by use of the Tegear energy operators. EEG waves are converted into 2D spectrum analyzer pictures to get textural details and classification criteria. The encryption spectrum analyzer picture of regular and seizure states was constructed to accurately measure the presented network efficiency on numerous encryption spectrum analyzer pictures. To encrypt the spectrogram picture, the Arnold and chaotic cryptographic techniques are used. Additionally, it is shown by the acquired visually encoded 2D spectrum analyzer pictures shown in Figure 4 that now the created pictures have commonalities and characteristics that are wholly separate from seizure and regular pictures, where each kind includes several distinct stripes. We were prompted to modify and apply the well-known deeper learning-based models, which was trained CNN structures for the seizure-specific diagnostic using our suggested approach because of the apparent disparities in the visualization properties obtained between regular vs. epileptic photos.

Encryption images were resized and transmitted to proposed fine-tuned CNN algorithms for automated extracting of the features, retraining, and classification. The encrypting routine and seizure photos must be scaled, but every CNN architecture has a different original size of the file periodicity, as indicated in Table 2. Secondly, the Encrypted EEG Spectrogram Classifier (E2SC) Module ‘transfer learning’ was used in this unit. Variables, weighting, and evaluation metrics from pre-learned CNN architectures that have been evaluated for computer vision applications using a generic image collection have been applied to the proposed malware image analysis problem that uses the Encryption EEG data repository. Ensemble learning was thus a successful approach for activity recognition evaluation in the system that was suggested in this study. Thus, we utilized AlexNet, Darknet-19, GoogLeNet, ResNet-50, and SqueezeNet to classify encrypted photos. Subsequently, swapping the last layers with fresh ones customized the newly encrypted dataset. It analyzes the actual number of courses (in our example two-classes) in learner visuals, automatically recompresses photos, sets parameters of the model, and trains the networks. Afterward, we classify test images to see if the system has been activated. 

#### 3.4.2. Module 2: Seizure Evaluation and Detection 

This section will evaluate the effectiveness of the offered strategy using different experimental settings.

#### 3.4.3. Evaluation of Fusion Properties Using Hybridized Spoofing Optimization

The hybrid seeks optimization method selects features and determines the ensemble classifier’s fusing variables t, r, and #. The resolution encoder provides the optimal fusion variables inside [0, 1] for improved precision. The gregarious and corvid searching agents’ traits are used in the suggested approach to tackle the variable selection and controller parameters scaling optimal issues. The proposed strategy improves local optimum and perfect global solutions by better connecting explorative and exploitative phases. Improving the real-world scalability problem requires multiple searching agents to choose the best site. 

#### 3.4.4. HSO Working Mechanism

Swarm intelligence (SI)-based solutions were the most effective way to solve worldwide optimization issues because of their variety, accessibility, and effectiveness. The SI approach to research approaches is unpredictable, unlike stochastic gradient descent. Regardless of their size, the corvid search agents are clever beings with enormous brains. They are more aware of themselves and can create tools. They recall faces and food locations for months. The corvid searching agents are known for their ability to form flocks, remember where food is concealed, follow one another while stealing it, and guard their young. The following is a discussion of these stages:

Step 1. Population configuration. The optimal solution, factors that have been identified, and restrictions are established. Gsize, flight duration, and Paw are also established.

Step 2. Memory/position normalization. The flock’s corvid search agents, Je, are initialized with e = 1, 2, ..., m. The searchers for corvids include placed at random. ZG I initializes corvid search agent memory.

Step 3. Evaluation of fitness resolution. The integrity of the location is assessed by substituting the selection parameters in the objective function to discover the fit measures in measures of accuracy.

Step 4. Creating a new role. The revised job description for the corvid iteration of the algorithm e is as follows:

(10) $$J_{CS}^{e,n+1}=J_{CS}^{e,n}+\lambda_{1}\times a^{e,n}\times \left(Z^{f,n}-J_{CS}^{e,n}\right)$$

*n*: iterative count, *λ*_1_ is the randomized integer between 0 and 1, and an is the flight duration. The approach is not preferred since there is a chance that the corvid searching agents will get stuck in the best local solution. In addition, the lower searching accuracy of the corvid candidate solutions must be improved; hence, the features of the gregarious candidate solutions are included in the suggested create a more personalized. The gregarious searching agents were chosen because of their adaptable uses in real-world engineering disciplines. The most significant trait that was inherited from the corvid iteration of the algorithm is its anti-predation traits. The modified description for the gregarious iteration of the algorithm is:

(11) $$J_{GS}^{e,n+1}=\left\{\begin{array}{ll} J_{best}^{n}+\theta \left|J_{GS}^{en}-J_{best}^{n,\partial }\right|, & \;\mathrm{if}\;j_{e}>j_{gl}\\ J_{GS}^{e,n}+\chi \left(\frac{J_{GS}^{EN}-J_{\mathrm{Uurd}\;}^{n}}{\left(j_{e}-j_{us}\right)+5}\right), & \;\mathrm{if}\;j_{e}=j_{gl} \end{array}\right\}$$

where the $\int_{\mathrm{best}}^{n}$ “*best*” is the gregarious searching agent’s global ideal location, *n*, is the step size, and *X* is a randomized number among −1 and 1. $j_{e}$ stands for the fitness of the current gregarious search agent, $j_{gl}$ for the globally best value, $j_{ws}$ for the globally worse resolution. Adding a new velocity-based variable:

(12) $$J_{\mathrm{best}\;}^{n,\partial }=J_{\mathrm{best}\;}^{n}+V^{n+1}$$

(13) $$J_{\mathrm{Hest}\;}^{n,\partial }=J_{\mathrm{best}\;}^{n}+\left(V^{n}+v_{1}\omega_{1}\times J_{\mathrm{best}\;}^{n}\right)$$

(14) $$J_{\mathrm{best}\;}^{n,\partial }=J_{\mathrm{best}\;}^{n}\left[1+v_{1}\omega \right]+V^{n}$$

(15) $$J_{GS}^{en+1}=J_{\mathrm{best}\;}^{n}+\theta \left|J_{GS}^{en}-J_{\mathrm{best}\;}^{n}\left[1+v_{1}\omega \right]+V^{n}\right|$$

Lastly, Ref. [16] hybridizes search agent choices dependent on corvid and gregarious attributes. The standard Equation (15) that include the uniqueness of corvid and gregarious evolutionary algorithms are employed in the suggested optimization procedure.

Step 5. If the positioning of the innovative mixture searching iteration of the algorithm cannot be made to work, the position will not be changed; in this case, the previous position will be maintained.

Step 6. Assessing your fitness for a new role. Re-evaluation of the fitness metric for the complete population of freshly created hybrid seeks search agents.

Step 7. Updating memories for a current career. Whenever the mixture engine seeks a local search that has higher fitness than just the previous one, its memories are modified as *J*^(*n*+1)^. The previous stages are continued until the terminal requirement is satisfied.

**Figure 5:**
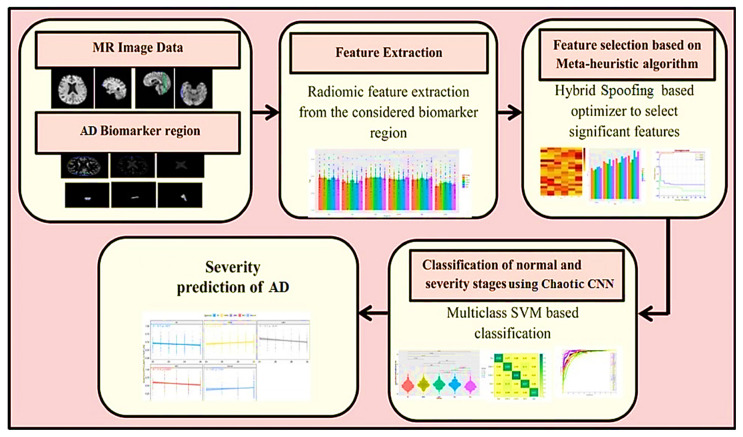
Stages of AD prediction.

Step 8. The approach includes identifying EEG signal characteristics as in Figure 5 and ensemble classifier hyper-parameters that can more effectively forecast the seizure disorder. The hybrid seeks optimization pseudo-code is provided in Algorithm 1.
**Algorithm 1** combines the traits of the gregarious pso algorithm with corvid searching characteristics.1: Input: $J_{i}^{G},i=\{1,2,\ldots ,m\}$ and G={1,2,…,W}
2: Outcome: $J^{n+1}$
3: Setting and loading the population of hybrid seeks search agent.4: Setting and initializing maximum iteration, I_max, flight length, Paw.5: Evaluate fitness values. 6: Modify the new position in accordance with Equation (15).7: Examine the viability.8: If (fitness old is lesser than fitness new).9: Re-evaluate fitness measure.10: Replace the old solution with the new one.11: Refresh memory.12: Return *J^n^*^+1^13: Terminate.

## 4. Experimental Results

Python, along with a large number of supplementary libraries, and in particular, the Keras computational intelligence API provided by the Pytorch machine-learning library, was utilized. While we are establishing our frameworks with external funding provided by Google Data with other data, a World Wide Web configuration continues to run on Google’s online storage. However, we have excluded the response time of building and implementing the analytical model as an indicator in our analyses. This is due to the variations in underlying infrastructure and GPU specifications, which can affect the accuracy of the results.

**Figure 6:**
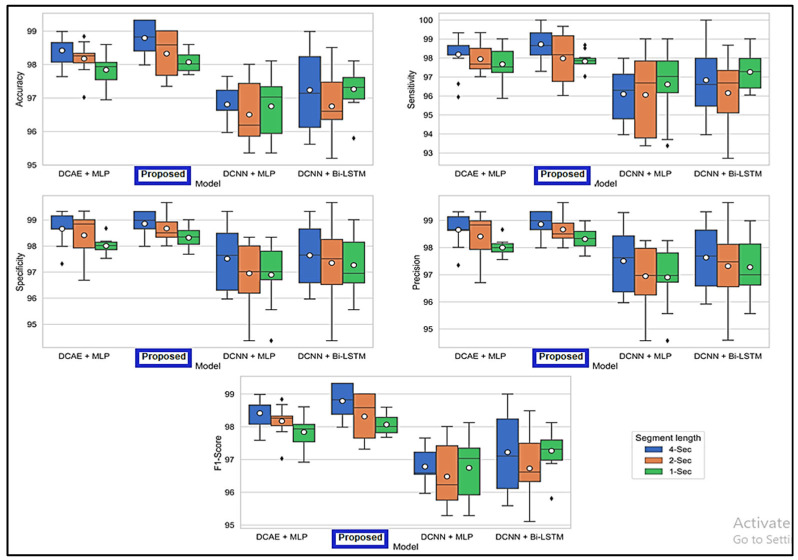
Boxplots displaying % values for evaluation metrics computed using the findings of the 10-fold crosses.

**Figure 7:**
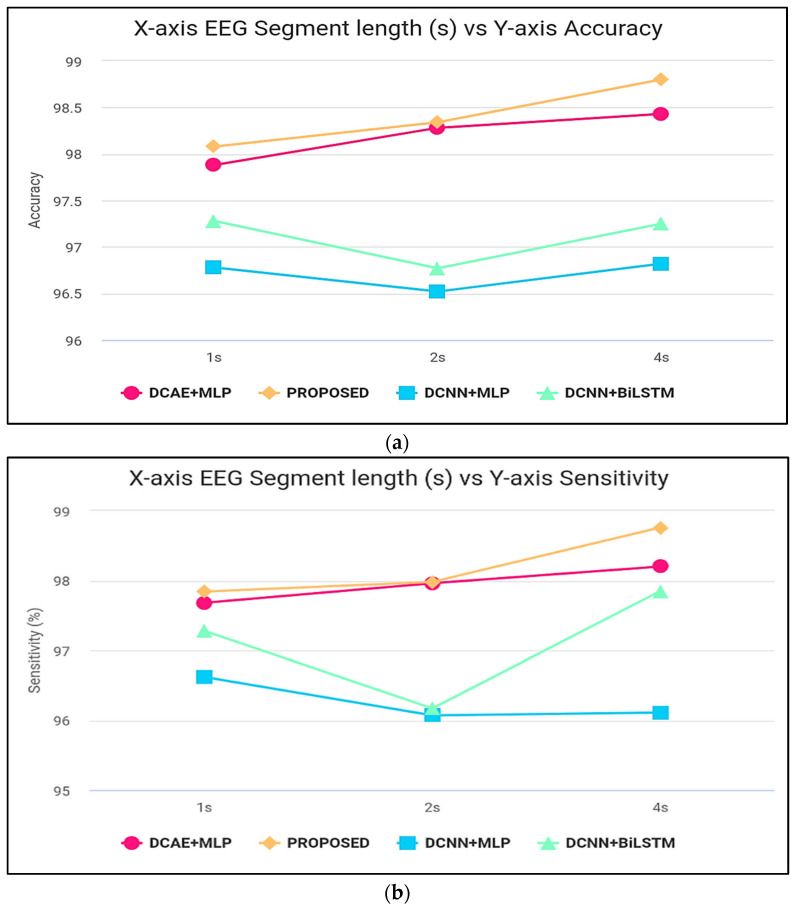

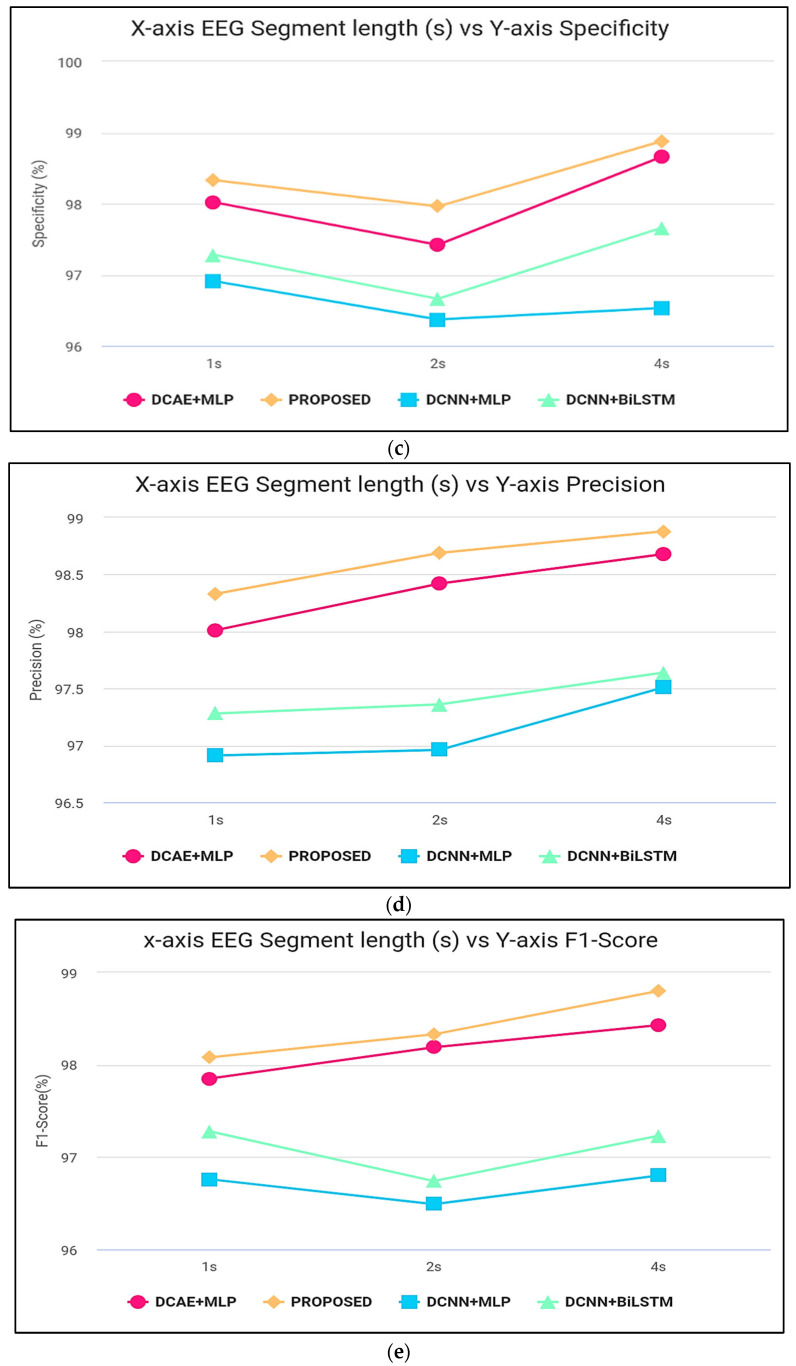
(**a**) Accuracy computation, (**b**) sensitivity computation, (**c**) specificity computation, (**d**) precision computation, and (**e**) F-score computation.

Figure 6 displays the ranges of parameters for the five performance benchmarks for every one of the four classifiers, and values were calculated using the 10-fold cross-validation classification precision of the devices with intervals of 1, 2, and 4 s. The findings provided in Table 3 are therefore shown as the median and the standard error for all measurements throughout the 10-folds. Figure 7 shows the same results. As shown by the findings, the two SDCAE algorithms beat the two non-AE algorithms (DCNN C MLP and DCNN C Bi-LSTM) for all EEG data streams and assessment metrics. As shown in Table 3, with a line length of 4 s, the PROPOSED model performed best in all rating systems. In all SDCAE algorithms, 4 s EEG sector duration yields the greatest classification performance. Overall, it is evident that all approaches that employed a Bi-LSTM for categorization outperformed their counterpart techniques that used MLP-based categorization for the same specific portion of the EEG. Bi-LSTM systems learn features faster than MLP models from low-dimensional space sequences. Lastly, the estimated coefficients in the analysis methods values for all findings indicate that SDCAE concepts have less variability than other variants, suggesting their efficacy is more steady over cross-validation repeats.

**Figure 8:**
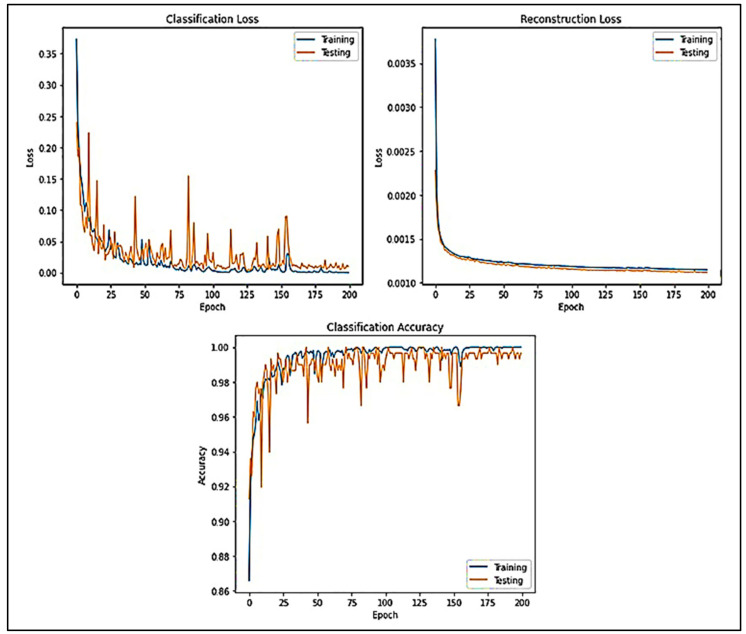
Accuracy and loss of data measured for various epochs.

Figure 8 displays the generalization of CLs and RLs lines of the datasets acquired while evolving the victorious network (DCAEmC Bi-LSTM) in one of the 10-fold cross-validity cycles. Then, classifying outcomes for the algorithms utilizing various EEG segment durations are presented in Figure 8.

### 4.1. Statistical Evaluation

The non-parametric Kruskal–Wallis H test tested the arithmetical power of categorization findings of the two suggested approaches. EEG segment lengths of 4 s were used to compare model assessment measures. The Kruskal–Wallis H test showed *p*-values of 0.0005% for accuracy, 0.02% for sensitivity, 0.025% for specificity, 0.05% for precision, and 0.001% for F1-measure while evaluating PROPOSED within the two approaches. Also, this analysis shows the *p*-values of 0.003 for accuracy, 0.011 for sensitivity, 0.083 for selective, 0.019 for precision, and 0.002 for F1-measure while evaluating DCAE C MLPs within the same two methodologies. All outcome evaluation criteria except specificity had *p*-values below 0.05. This demonstrates the differences in significance level between the results of all proposed frameworks.

### 4.2. Similarity with Other Techniques

The literature utilizes several measures to evaluate seizure categorization systems. This chapter will solely compare measures like accuracy, responsiveness, and specificity. Table 4 compares the performance of our special edition to a few cutting-edge deep neural network algorithms for extracting the features and seizure categorization. In [31], many stacked sparse noise removal auto-encoders (SSDAE) were evaluated for quality extraction and connection among STFTs. Using a random assortment of train and test sets, they achieved the best accuracy of 93.82%. The authors of the 2018 study [32] integrated the global maximum MICs with the VGGNets for extraction and classification of features. They obtained 98.2% correctness, 98.86% sensitivity, and 97.48% selectivity using fivefold cross-validation [33] classified patient-specific epileptic and interictal events using FFTs for frequency domain estimation with CNNs. 

**Figure 9:**
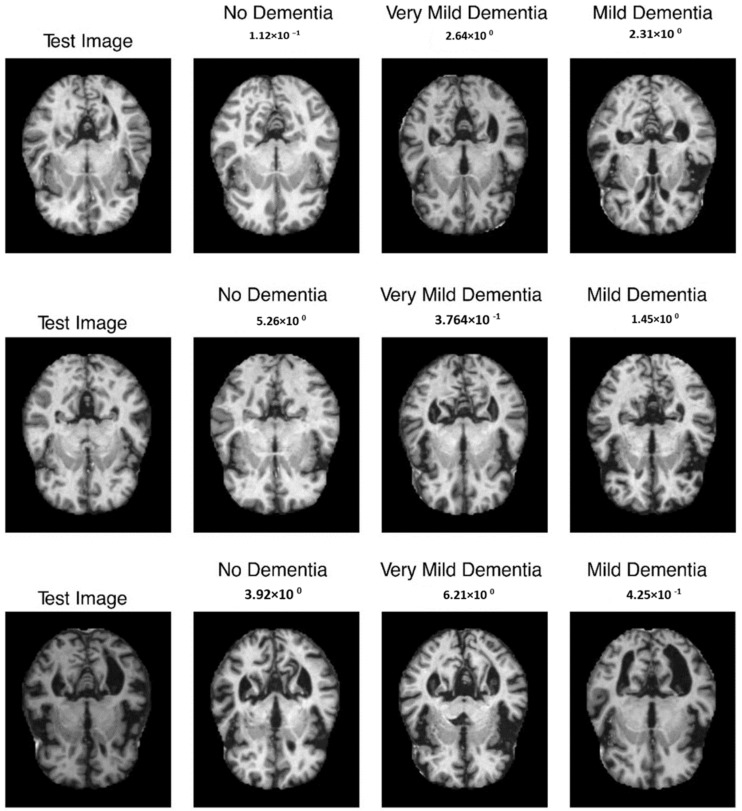
Sample output with absence, very mild, and mild dementia.

The mean evaluation methods for all patients included 97.5 percent reliability, 96.4 percent sensitivity, and 98.1 percent specificity after sixfold cross-validation. Last but not least, Refs. [34,35] used 2D-CNN techniques to predict both spatial and temporal features of EEG records were used for patient-specific authentication using randomized evaluation metrics. They obtained 91.6 percent selectivity, 90 percent sensitivities, as well as 98.05 percent correctness for the cross-patient information. The sample outcome is shown in Figure 9.

**Figure 10:**
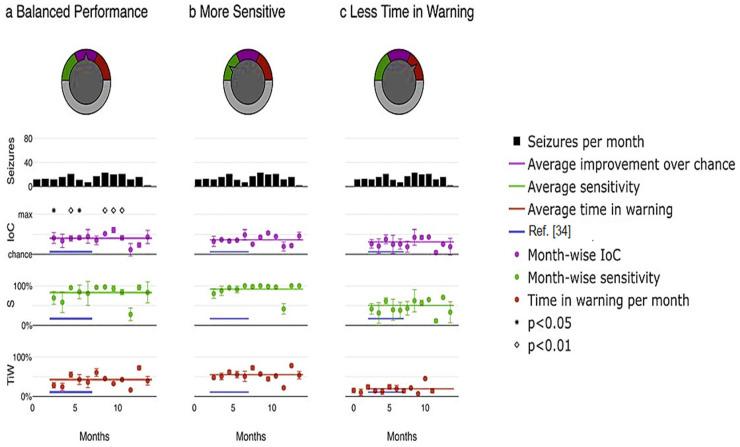
Pseudoprospective study on seizure prediction. (**a**) prioritizes balanced performance, (**b**) prioritizes higher sensitivity, and (**c**) prioritizes minimizing time in warning.

Furthermore, the proposed model enables the customization of the system based on the preferences of physicians or patients by adjusting the relative emphasis on sensitivity and time in warning. Figure 9 illustrates an example application of this adaptable system. Different priorities were selected, including maximizing the improvement over chance (Figure 10a), placing greater importance on high sensitivity by assigning a weight of three to sensitivity compared to time in warning (Figure 10b), and minimizing the duration of the warning state by assigning a weight of three to time compared to sensitivity in warning (Figure 10c). Through a user-friendly interface, a single model parameter can be modified, allowing patients or physicians to easily determine which metric to prioritize and to what extent in real-world scenarios, as depicted in Figure 10.

## 5. Conclusions

To diagnose seizures in pediatric patients, a unique deep-learning technique is suggested. By categorizing slightly pre-processed raw wideband EEG signal recording, the unique technique employs a chaotic CNN to identify epileptic episodes. An HSO is utilized to categorize epileptic or intricate brain state EEG data to take advantage of its high-efficiency automated feature learning and classifying. Three different EEG data section durations were employed to construct and evaluate two different chaotic models. These models make use of the baker map and HSO-trained network-based classifications. To compare the outcomes of the two suggested algorithms to two conventional deep learning techniques that have the same layered but no decoding infrastructure layer, the 12 templates were developed and assessed to use a tenfold cross-validation approach, and the CNNs design that makes use of chaotics within 4 s EEGs portions, was the framework with the highest points results. This algorithm has a final F1-score of 98.80% and averages 98.80% correctness, 98.74% sensitivities, 98.87% selectivity, and 98.82% precision. This chaotic CNN with HSO algorithm outperforms most state-of-the-art techniques with diverse datasets. In futuristic work, the same CNN architecture with chaotic will be extended for multi-modal inputs from various sources. While the available information might not provide a comprehensive overview of the clinical data, recognizing these limitations is crucial for accurately interpreting the study’s results. It is essential to weigh the potential impact of these factors to the study’s conclusions and to encourage future research endeavors that could address these limitations through larger and more diverse sample populations. This could ultimately enhance the reliability and applicability of the study’s findings within broader clinical contexts.

## Figures and Tables

**Table 1:** Summary of ML algorithms efficacy from previous researches.

Ref. No.	No. of Samples	No. of Seizures	No. of Features	EEG Signals	Average Sensitivity %	FPR*h*^−1^
[18]	6	86	3	2/3	88	0.15
[17]	24	87	12	6	73.98	0.06
[15]	10	86	22	6	71.97	0.17
[19]	20	86	18/23	18/23	88.34	0.155
[16]	24	87	22	6	73.08	0.33

**Table 2:** The CNN models’ picture dimensions.

Design	Size of Image
AlexNet	227 × 227
Darknet-19	256 × 256
GoogLeNet	224 × 224
ResNet-50	224 × 224
SqueezeNet	227 × 227

**Table 3:** Shows the classifier outcomes for various EEG particular sections.

Length of EEGs Segment.	Methods	Accuracy in %	Sensitivity in %	Specificity in %	Precision in %	F1-Score in %
1 s	DCAE + MLPs	97.85 ± 0.45	97.67 ± 0.88	98.67 ± 0.88	97.67 ± 0.76	97.67 ± 0.89
	PROPOSED	98.09 ± 0.32	97.88 ± 0.59	97.45 ± 0.84	95.67 ± 0.88	97.77 ± 0.67
	DCNN + MLPs	97.66 ± 0.89	94.67 ± 0.84	97.66 ± 0.76	97.87 ± 0.86	96.67 ± 0.78
	DCNN + Bi-LSTMs	96.68 ± 0.49	97.67 ± 0.86	95.67 ± 0.88	96.67 ± 0.88	98.67 ± 0.78
2 s	DCAE + MLPs	97.68 ± 0.73	96.67 ± 0.88	97.77 ± 0.78	98.67 ± 0.88	98.67 ± 0.98
	PROPOSED	97.53 ± 0.96	97.65 ± 0.78	97.57 ± 0.88	98.67 ± 078	97.77 ± 0.86
	DCNN + MLPs	96.69 ± 1.03	97.66 ± 0.88	96.67 ± 0.78	97.67 ± 0.76	97.67 ± 0.78
	DCNN + Bi-LSTMs	95.57 ± 0.78	97.67 ± 0.67	97.68 ± 0.76	96.67 ± 0.88	95.67 ± 0.88
4 s	DCAE + MLPs	95.72 ± 0.86	95.67 ± 0.88	96.66 ± 0.88	96.67 ± 0.78	96.97 ± 0.88
	PROPOSED	98.67 ± 0.67	97.68 ± 0.85	97.67 ± 0.78	98.67 ± 0.78	96.68 ± 0.88
	DCNN + MLPs	98.47 ± 0.79	97.67 ± 0.82	97.67 ± 0.72	98.67 ± 0.88	99.67 ± 0.78
	DCNN + Bi-LSTMs	95.68 ± 0.67	97.67 ± 0.75	98.74 ± 0.67	96.67 ± 0.78	95.67 ± 0.78

**Table 4:** Comparison of metrics vs. datasets vs. techniques.

Extraction of Features	Collection of Dataset	Accuracy in Percentage	Sensitivity in Percentage	Specificity in Percentage
STFT + SSDAEs	Randomly	94.83%	N/A	N/A
MIC + VGGNets	5-FoldCV	98.2%	98.87%	97.48%
FFT + CNNs	6-FoldCV	97.6%	96.87%	99.16%
2D-CNNs	Randomly	98.06%	91%	92.66%
PROPOSED	10-FoldCV	98.80%	98.73%	99.87%

## Data Availability

Data will be available on request.

## Abbreviations

2D-CNNs	2-Dimensional Convolutional Neural Networks
Bi-LSTM	Bidirectional Long Short-Term Memory
CNN	Convolutional Neural Network
DCNN	Deep Convolutional Neural Network
DL	Deep Learning
EEGs	Electroencephalograms
F1-Score	F1-Measure
G	Set of values or indices
GPU	Graphics Processing Unit
HSO	Hybrid Seek Optimization
I_max	Maximum number of iterations
LSTM	Long Short-Term Memory
MIC	Maximal Information Coefficient
MLP	Multi-Layer Perceptron
N × N	N by N (matrix dimension)
Paw	Population Average Weight
PRNG	Pseudo-Random Number Generator
SDCAE	Stacked Denoising Convolutional Autoencoder
SSDAE	Stacked Sparse Denoising Autoencoder
STFT	Short-Time Fourier Transform
TKEO	Teager–Kaiser Energy Operator
W	Set of values or indices
